# Revisiting critical STEM interventions: a literature review of STEM organizational learning

**DOI:** 10.1186/s40594-022-00357-9

**Published:** 2022-06-17

**Authors:** Norma López, Demetri L. Morgan, Quortne R. Hutchings, Kendrick Davis

**Affiliations:** 1 Institute for Democracy and Higher Education, Tufts University, Medford, USA; 2 Department of Higher Education, Loyola University Chicago, Chicago, USA; 3 Department of Counseling and Higher Education, Northern Illinois University, DeKalb, USA; 4 Center for Race and Equity, University of Southern California, Los Angeles, USA

**Keywords:** Change processes, Organizational learning, STEM education, URM students

## Abstract

There is inconclusive evidence on the ability of scientific research in science, technology, engineering, and mathematics (STEM) education to scale-up from one context to another and ultimately become institutionalized. The dearth of evidence draws focus on how organizations change and evolve or the process of organizational learning. We designed this systematic review of the literature to uncover to what extent and how organizational theory has been leveraged within STEM interventions or as a research tool to inform the policies and practices of STEM education organizations. Unlike previous reviews, we explicitly focused on how organizational learning informs cultural transformation toward the success of racially and ethnically underrepresented minority (URM) students in STEM. The research question was: How has organizational theory and learning informed the potential for STEM education to center the success of URM students? Our results reveal that STEM entities that did not leverage organizational theory consistently fell into either the “decision trap” identified by Langely et al. created by ignoring temporal structures or deemed the innovation threatening, as Kezar suggested. We conclude with practical recommendations for the design of STEM education interventions.

## Introduction

A defining feature of the Louis Stokes Alliance for Minority Participation (LSAMP), a signature National Science Foundation (NSF) program, is contributing to the success of underrepresented minorities (URMs) in science, technology, engineering, and mathematics (STEM). LSAMP initiatives promote collaboration among institutions in a geographic region to achieve collaborative outcomes for racial and ethnic URMs in STEM (James & Singer, 2016). Specifically, the LSAMP program provides support to URM students in STEM by establishing mentoring and research opportunities and implementing pedagogical innovations in STEM classrooms that promote student-centered learning. The goal of the LSAMP initiative is that, eventually, the programs and knowledge produced by the alliances become institutionalized, self-perpetuating, and self-supported. To spur this outcome, LSAMP programs funded for ten consecutive years, when applying for new funds, are required to

address the institutionalization and sustainability of LSAMP-supported activities by stating the progress they have made towards sustainability. They must also detail the institutionalized components and describe any systemic changes in STEM departments or alliance institutions that have resulted from the NSF LSAMP investment. (NSF, n.d.-b, p. 7)

Additionally, LSAMP programs must promote knowledge production and dissemination. We are part of the research team on the Illinois LSAMP. This systematic review of the literature was prompted by the dearth of published literature at the intersection of STEM education research and organizational theory to illuminate the role of organizational learning in institutionalization of URM success. Furthermore, though the Urban Institute concluded that alliances should seek to *replicate* the LSAMP framework based on the identified characteristics (Clewell et al., 2006), the report authors provided little guidance on the best strategies to encourage the process of scaling-up the identified successful components of the model from one context to another, such as mentoring, undergraduate research, and professional development for faculty. Nor is there any guidance on what institutions should or could be doing to make LSAMP programs sustainable if federal funding were to cease or critical personnel was no longer affiliated with the project (i.e., the process of institutionalization).

To understand and guide organizational learning, scholars often turn to insights from organizational theory to help provide awareness of how people interact with, shape, and become influenced by the culture, norms, and policies of an entity (Bastedo, 2012; Birnbaum, 1989; Bolman & Deal, 2017). Popova-Nowak and Cseh (2015) defined organizational learning as “a social process of individuals participating in collective situated practices and discourses that reproduce and simultaneously expand organizational knowledge… that results in adapting to the environment” (pp. 316–317). Scholars have leveraged organizational theory to explore a diverse array of topics, including faculty socialization (Gonzales, 2018; Tierney, 1997), institutional dynamics related to equity and inclusion initiatives (Baker & Blissett, 2018; LePeau et al., 2016), and civic engagement efforts (Barnhardt, 2015; Morgan, 2019).

Building on these studies, the purpose of this systematic literature review was to interrogate how organizational theory has been leveraged within STEM interventions or as a research tool to inform the policies and practices of STEM education organizations that advance cultural transformation toward the specific success of racially and ethnically URMs in STEM. Due to the lack of research specifically focused on organizational theory related to URM STEM institutionalization efforts, we focused this systematic literature review on both STEM interventions that specifically target URM populations and STEM interventions that LSAMP is implementing to diversify the STEM pipeline (e.g., research and mentoring opportunities, curricular innovations, etc.). The guiding question for this study was: How has organizational theory and learning informed the potential for STEM education to center the success of URM students?

## Literature background

The scant research at the intersection of STEM education and scale-up efforts required the inclusion of a combination of organizational literatures. Two considerations that guided this literature review were the collective nature of LSAMP programs and the assumption that innovation, once tried and tested, is beneficial in any context (Kezar, 2011).

Given the inherently relational nature of the LSAMP model and other STEM networks, one way to situate our study was to leverage an ecological approach to understanding organizations. A tenet within the ecological lens is that organizations change in response to their environments (Hannan & Freeman, 1977). Hannan and Freeman (1984) distinguished ecological changes within organizations from adaptive change processes by noting that adaptive mechanisms to change face numerous limitations, including structural inertia, historical legacies, and political constraints. Therefore, a more precise and relevant way to explain or generate change is to ascertain how an entity is positioned relative to similar entities. To this end, organizational ecology researchers invoke the concept of isomorphism to describe why organizations change in response to internal and external environmental factors (Fumasoli & Stensaker, 2013). Isomorphism has been broadly defined as a “constraining process that forces one unit in a population to resemble other units that face the same set of environmental conditions” (DiMaggio & Powell, 1983, p. 149)

Kezar (2011) highlighted that many scaling-up efforts in educational organizations had not reached successful replication because the efforts "often involve a static innovation that is considered to work in different contexts, even as circumstances change over time” (p. 240). Institutionalization and scaling-up efforts may be limited because scholars and educators do not attend to the dynamic organizational features that encompass educational interventions designed to promote the success of URMs in STEM. The following section situates our study in a broader body of knowledge related to organizational change efforts and the diffusion of ideas as one avenue to conceptualize organizational change processes related to efforts to realize broader impacts within STEM education research.

### Isomorphism

DiMaggio and Powell (1983) extended the concept of isomorphism to focus on institutional isomorphism, which they argued acclimates researchers to how “organizations compete not just for resources and customers [students], but for political power and institutional legitimacy, for social as well as economic fitness” (p. 150). Isomorphic change occurs in three ways: (a) coercive isomorphism, of political or administrative pressure for change; (b) mimetic isomorphism, or the pressure to change in light of ambiguous circumstances; and (c) normative isomorphism or change associated with the expansion of training and education as well as the diffusion of ideas through a network.

Many higher education scholars have leveraged this framework to study different dimensions of institutional change efforts (Garcia, 2017; Pusser & Marginson, 2013; Slaughter & Rhoades, 2004). Nevertheless, these studies did not establish how institutional isomorphism operates more collaboratively and synergistically. Much of the ecological and isomorphism research on institutional change presupposes a lack of resources, competition from similar entities, bureaucratic impediments, and a dearth of consensus around goals. Isomorphism researchers have clarified the need to identify and address factors in the environment that surround STEM entities in the organizational learning processes. The following section highlights organizational dynamics within a network that complement the external focus of isomorphism.

### Social cognition

Over the years, scholars have explained the need to embed individual cognitive processes within organizational dynamics to account for characteristics like an entity’s organizational culture, which simultaneously shapes and is shaped by individuals in the organization (Allard-Poesi, 2005; Bolman & Deal, 2017). Given the relational nature of LSAMP programs, we looked to Akgün and colleagues (2003) 10 distinct but related factors that constitute an intra-organizational learning process:

(1) information acquisition; (2) information implementation; (3) information dissemination; (4) unlearning (i.e., discarding information); (5) thinking (i.e., manipulation of memory); (6) intelligence (i.e., ability and capability to process information); (7) improvisation (or autonomous behavior) (i.e., learning with actions or reflection); (8) sensemaking (i.e., giving meaning to information); (9) emotions; and (10) memory (i.e., information storage). (p. 844)

These features relate to Birnbaum’s (1988) foundational work in higher education governance that focused on cybernetic, or self-correcting, processes within institutions. Birnbaum’s work built on Weick’s (1976) idea that higher education institutions are unique in their organizational design in that numerous organizational cultures can be present at any given moment. Thus, to manage change and lead these organizations well, Birnbaum (1989) called for administrators to engage in organizational learning by using “multiple frames to develop richer behavioral repertoires, increase[ing] the sensitivity of institutional monitoring systems, and focus[ing] attention on important issues through systems that report data and create forums for interaction” (p. 239).

### Summary

With this brief review, we tried to make evident how the isomorphism literature highlights the external environment as the main issue when seeking to understand organizational change processes within a network. However, the literature is not nuanced enough to highlight intra-organizational change dimensions, hence our focus on social cognition. Ultimately, institutions are complex social organizations, and numerous factors contribute to organizational learning. Our research will contribute to the extant literature by providing a framework that URM STEM alliances could utilize in categorizing and implementing an intentional change process. We now turn to our theoretical framework designed to help braid the isomorphic and social cognition bodies of knowledge together to help unearth the intersection between STEM education empirical studies and organizational learning.

## Theoretical framework

Fiol and Lyles (1985) distinguished between two aspects of organizational learning: acquiring new awareness or knowledge and creating new systems or structures. Specifically, “organizational learning means the process of improving actions through better knowledge and understanding” (Fiol & Lyles, 1985, p. 803). Fiol and Lyles recognized that change is not always about organizational learning but can be attributed to “defensive adjustment” (p. 805). Specifically, defensive adjustment refers to not understanding causal relationships. Therefore, our theoretical framework draws on Langley et al.’s (2013) categorization of change studies of organizations, which characterizes new insights, and Kezar’s (2011) three mechanisms for scaling-up outcomes from NSF-funded work, which illustrates systems or structures. In other words, Langley and associates work allowed us to focus the analysis on the dynamic nature of and motivation for change. In contrast, Kezar’s research drew our attention to the systems and procedures needed for that change.

### Langley’s categorizations

Langley et al. (2013) reviewed responses to a call for papers focused on addressing questions “about how and why things emerge, develop, grow, or terminate over time” (p. 1). Of relevance to us, they intently focused on temporality as a way to operationalize the environmental realities organizations face as they change. Put in their terms:

Knowing that organizational practice B is generally more effective than organizational practice A reveals almost nothing about how to move from A to B. Moreover, depending on the nature of the practices concerned and the context of their application, it could be that the very process of moving between A and B itself engages resources, political dynamics, and organizational upheaval that could place the original evidence supporting the need for change in an entirely different light. (Langley et al., 2013, p. 4)

The first concept is the ontological stance one takes in unpacking change processes. Langley et al. argued that change is either conceptualized as a successive progression with an outcome (e.g., You do X until you achieve Y) or a never-ending process (e.g., You must keep doing X while moving closer to realizing Y). The latter focuses on changes in processes, whereas the former focuses on the change in things. As a result, trying to distill the ontological underpinnings of the studies is critical in conceptualizing the ecosystem of organizational theory and STEM. The second concept is the idea of tensions or paradoxes in change processes rather than change described as a cycle or linear. Focusing on how entities wrestle with obstacles helps highlight potentially transferable aspects to other contexts. Finally, Langley et al. described the notion of stability in terms that are dynamic to account for the “active work [that] is required to maintain practices, organizations, and institutions” (p. 10). This claim highlights the importance of studies to address the aftermath of change and how entities can maintain new realities while evolving.

### Kezar’s mechanisms

Regarding the mechanisms for scaling-up change efforts in an organization, Kezar (2011) first highlighted nine persistent critiques of scale-up efforts from the literature: incentives, implementation context/flexibility, depth, ownership, underlying norms, sustainability, spread, static nature, and motivation. Kezar highlighted deliberation, networks, and external support and incentives as the main levers to facilitate lasting change or operationalize institutionalized change. Deliberation refers to “a process whereby people come to an understanding and learn” (Kezar, 2011, p. 242). Deliberation draws attention to an interactive process rooted in dialogue and the affirmation of one’s voice in the change process. On the other hand, networks draw attention to the flow of information that spurs adaptation, as “networks connect people to others with similar ideas and also provide change agents with the information needed to help move the change process along” (Kezar, 2011, p. 242). Finally, external supports and incentives refer to “the funding, awards, and recognition necessary in order to help sustain change agents in the face of entropy and even negative dynamics” (Kezar, 2011, p. 243). Kezar’s three mechanisms are all predicated on an organizational learning perspective of change and draw attention to specific dimensions of how individuals within an organization position themselves to approach their work.

### Six dimensions of organizational change in STEM

Building on the reviewed literature and grounded in the two frameworks presented above, our operating proposition is that studies must address six dimensions of organizational change. Table 1 provides an overview of the dimensions and main questions we leveraged when operationalizing the framework.

**Table 1:** Organizational change in STEM

Dimensions	Key question
Intentional ontological stance on change	Does the study make explicit how they understand the process of change?
Identification of tensions and paradoxes	Does the study identify points of tensions or paradoxical experiences within the change process?
Stability described as an on-going process	Is the stability of the change process addressed?
Deliberation among stakeholders	Is there evidence that the change process involves deliberation among stakeholders?
Expansion of social networks	Does the change process involve expanding the stakeholder’s social network?
Timely access to support and/or incentives	Does the change process described narrate timely access to modes of support or incentives?

**Fig. 1:**
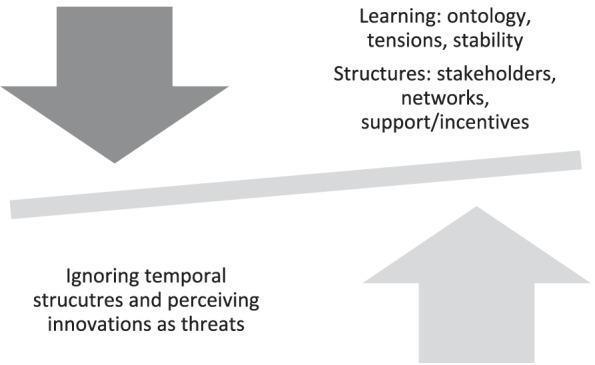
Dimensions of organizational change

Combining these frameworks highlights the knowledge and structures needed for scaling-up efforts and exposes tensions within each framework that can lead to failure to change. Langley et al.’s (2013) conceptualization emphasized the importance of time and points to how ignoring temporal structures, for example, “how decisions that looked good at one time turn catastrophic at another” (p.4), can cause innovations to falter or fail. Kezar (2011) further notes that not only are innovations “inherently context based” (p. 239), but they also have to adapt to the innovation to reflect their context; without these measures, innovations that are externally introduced are perceived as threats to internal interests and resources. The figure below captures the dynamic nature of our framework to better orient the reader to the harmony and tensions created by the combination (Fig. 1).

## Research design

Tranfield et al. (2003) noted systematic reviews of literature (SRLs) differ from traditional narrative reviews “by adopting a replicable, scientific and transparent process... by providing an audit trail of the reviewer’s decisions, procedures and conclusions guidelines” (p. 209). Specifically, we leveraged Rew’s (2011) 13-step process for clarity and concern translating research insights into practice. The first two steps in the process are identifying a research question to be answered and stating the purpose of the review, both of which we did in the introduction.

### Setting up the SRL

#### Inclusion criterion

The third step entails stringent and clearly defined inclusion criteria based on the research question (Rew, 2011). Using the collective insight of members of the research team (Norton, 2008), we focused database construction around articles that explicitly dealt with concepts of sustainability of educational interventions or taking educational interventions to scale in STEM––meaning how educational interventions go from one organizational context to another. By educational interventions, we mean initiatives identified in the Urban Institute report (Clewell et al., 2006), such as mentoring, undergraduate research, internships, and academic advising. This approach builds on but is different from Henderson et al.’s (2011) review of change efforts within a unit or program. Finally, given the research question, we narrowly focused on empirical studies, meaning we only included studies that had articulated research questions, study designs, and findings in the final analysis.

#### Search terms

Step 4 calls for demarcating search terms (Rew, 2011). We used STEM in different configurations accompanied by other concepts such as “organizational theory”, “curricular reform”, “academic advising”, and “research.” We used these terms as LSAMP's innovations seek to achieve URM STEM success through targeted programs focused on curricular reform, creating research opportunities, and providing advising through this additional resource. We also used terms such as “critical” and “bias” to capture articles focused on people of color in STEM.

#### Identifying databases

Step 5 calls for identifying the appropriate databases to search within. Following other higher education SRLs (Harper, 2012; Mitchell et al., 2014; Renn, 2010), we decided to focus on STEM education and higher education research journals (see Table 2 for a list). We delimited the search timeframe in these repositories to articles published since 1970 when calls for broadening participation in STEM commenced (James & Singer, 2016).

**Table 2:** List of target journals

Journals
Administrative Science Quarterly Advances in Engineering Education AERJ
AGB Library/Reports Change
Community College Journal of Research and Practice Community College Review
Educational Researcher Harvard Educational Review Higher Education
Higher Education Quarterly
International Journal of Engineering Education International Journal of Seine Education International Journal of STEM Education Journal for STEM Education Research
Journal of College Science Teaching Journal of College Student Development
Journal of Computers in Math and Science Teaching Journal of Diversity in Higher Education
Journal of Engineering Education
Journal of Excellence in College Teaching Journal of Higher Education
Journal of Research in Science Teaching Journal of Research in STEM Education Journal of Science Education and Technology
Journal of STEM Education: Innovations and Research Journal of Student Affairs Research and Practice Mathematics and Computer Education
Nature
Proceeding of the National Academy of Sciences Research in Higher Education
Russell Sage Journal of the Social Sciences Science
Science and Education Science Education Teachers College Record
The Review of Higher Education

### Data collection in an SRL

Steps 6–9 relate to searching and include specifying and reviewing the search outcomes to match the inclusion/exclusion criteria, systematically extracting data, and determining the quality of the reviewed studies. A research team member was assigned a series of journals to review. After setting the search parameters to match the inclusion/exclusion criteria, a team member read the title and abstract of any study that came up. If it was deemed worthy of inclusion in the database, the following pertinent study information was captured in an online form:

- Title of Journal
- Name of Article
- Year Published (1970–Present)
- What are the study’s research questions or hypothesis?
- What is the study’s approach to data analysis?
- What is the focus of the article? (Type of STEM Educational Intervention)
- What is the unit of analysis? (Who is the study about?)
- Is there an explicit mention of organizational theory in the literature review or theoretical framework?
  o If yes, what organizational theory is mentioned?

Using this process, we captured 24 manuscripts. We also reviewed Henderson et al.’s (2011) publicly available database from their literature review to see if we missed any studies that we could include but perhaps were not in our initially identified journals. We identified three additional studies from their database, added the journals to our list, and reviewed the journals to ensure no new studies fell outside the 2008 timeframe of the Henderson et al. database. In sum, 27 manuscripts met our inclusion criteria and were relevant to our research question. In step 10, we compiled critical information and the findings from each study into a table to prepare for analysis (Rew, 2011).

### Analytical plan

The 11th consideration is a straightforward procedure for analysis (Rew, 2011). Phase 1 of our analysis included dividing the studies between two team members and descriptively categorizing the studies into groups based on the methodological approach, level of analysis (student, faculty, or institution), and whether an explicit organizational theory guided the work. The second author reviewed each of the categories. In the few cases where there was a discrepancy in the study’s categorization, the research team reached a consensus on how to code the study (Tracy, 2010).

Phase 2 of the analysis leveraged our dimensions of organizational change in the STEM framework (Table 1) as deductive codes (Saldaña, 2016) to explore how and to what extent the literature connects to these dimensions. In this phase, two team members reviewed each study again, and portions of the study were assigned codes based on the six dimensions. The first three authors then met to engage in a constant comparison discussion to explore nuances that emerged within each dimension connected to examples from the research articles (Saldaña, 2016).

In Phase 3, the research team distilled themes from the emerging nuances and crafted descriptive summaries of how the different studies engaged the dimensions of the theoretical framework.

## Limitations and trustworthiness

The 12th step of Rew’s (2011) SRL acknowledges any inherent limitations and biases in the process.

### Source limitations

A significant point of caution is that STEM studies exist in various venues and formats that we did not include in the corpus of research journals. Therefore, some studies may be relevant to the research question that we did not assess. Future researchers should expand the venues and types of formats included in the corpus to include books, monographs, reports, and website resources.

### Theory limitations

Another limitation is that we focused narrowly on two related aspects of organizational learning, scaling-up interventions and making interventions institutionalized, to the exclusion of the entire change process. As a result, our analyses missed important precursors and more person-centric (e.g., identity-related) factors that are critical in both social cognition and isomorphic processes.

### Identifying and addressing bias

In terms of biases, we followed suggestions in making plain the seen, unseen, and to the best extent, unforeseen issues in our collective approach to the topic (Milner, 2007). Our research team is diverse across race (e.g., Black and Latinx), ethnicity (e.g., Mexican), gender identity (e.g., cis-males and cis-female), sexuality (e.g., Queer), positioning in and outside the academy (e.g., faculty member, graduate students, non-profit think tank), and affiliation with the STEM community (e.g., former engineer, former STEM academic advisor, STEM mentor). These multiple vantage points meant we interacted with and coded the studies in our database differently. We resolved disagreements through critical inquiry dialogues among the team members participating in the data analysis (Ravitch & Carl, 2016). Nonetheless, our positionality and social locations informed our decisions throughout the study. Consequently, though we made evident the steps we took to conduct the SRL, researchers with different identities may reach different conclusions.

### Enhancing credibility

To strengthen the study's trustworthiness, we addressed different aspects of Tracy’s (2010) “big-tent” criteria for excellent qualitative research. To enhance the credibility of the research, the fourth author reviewed, probed, and critiqued the data analysis and presentation of the findings to support our effort to “crystalize” the present understanding of organizational learning in STEM education. Tracy noted that the goal of crystallization is to “open up a more complex, in-depth, but still thoroughly partial, understanding of the issue” (p. 844). We enhanced sincerity through our transparent account of how our theoretical framework informed our research design and how we sought to carry out the research design with authenticity by remaining open to how our narratives informed our analysis and interpretation of the data. Finally, we sought resonance by (re)presenting the results to highlight transferable findings in future research and implications for policy and practice––aided by thick descriptions of themes with concrete and representative examples from the studies in the database (Tracy, 2010).

## Results

We first present the descriptive results of our SRL to provide a sense of the types of studies in the database. Table 3 summarizes the databases' studies by their approach to research and presents a representative study from each category. Research approaches included qualitative research, quantitative research, literature/discourse analysis, and mixed/multiple methods. Table 4 highlights the unit of analysis and research questions and leverages a different set of illustrative studies. Units of analysis included studies concerned with the entire field of STEM education, the college/university as an entity, programs/departments, or students. Table 5 showcases categories that focused on different types of explicit organizational theories used in the studies in the database.

**Table 3:** Research design examples

Category	*n*	Example study	Approach to research
Literature review/ Discourse analysis	6	Inside the double bind: A synthesis of empirical research on undergraduate and graduate women of color in science, technology, engineering, and mathematics (Ong et al., 2011)	Literature review
Mixed/Multiple methods	3	Closing the Gaps and Filling the STEM Pipeline: A Multidisciplinary Approach (Doerschuk et al., 2016)	Questionnaires; interviews; secondary institutional data
Qualitative methods	5	The intersectional matrix: Rethinking institutional change for URM women in STEM (Armstrong & Jovanovic, 2017)	Document analysis and interviews
Quantitative methods	13	Beyond Traditional Measures of STEM Success: Long-Term Predictors of Social Agency and Conducting Research for Social Change (Garibay, 2018)	Multilevel modeling

**Table 4:** Unit of analysis and research question examples

Category	*n*	Example titles	Research question/Hypothesis
Colleges/University/Field	3	A Systems Model of Innovation Processes in University STEM Education (Porter et al., 2006)	Whether innovation models and experiences from outside the education arena could help elucidate educational innovation processes?
Department/School	2	Assessing Institutionalization of Curricular and Pedagogical Reforms (Colbeck, 2002)	What do participants in a reform effort consider are good indicators of lasting change?
Faculty	4	The Roles of STEM Faculty Communities of Practice in Institutional and Departmental Reform in Higher Education (Gehrke & Kezar, 2017)	How are faculty engagement in and perceptions of cross-institutional CoPs’ design characteristics associated with local institutional and departmental change related to STEM reform after controlling for institutional, professional, and personal characteristics?
Program	3	Institutional Sources of Practice Variation: Staffing College and University Recycling Programs (Lounsbury, 2001)	How broader institutional dynamics driving the diffusion of recycling got translated into more specific staffing arrangements in college and university recycling programs: whether newly adopted recycling programs at colleges and universities were staffed by existing employees who assumed recycling duties as an additional responsibility (role accretion) or by full-time employees who filled newly created roles in the organizational chart (status creation)
Students	5	Utilizing factor analysis to inform the development of institutionally contrived experiences to increase STEM engagement (Morgan & Gerber, 2016)	What institutionally contrived experiences can be implemented to increase STEM skills (e.g., analytical, organizational, mathematical, technical, problem solving, and communication skills), knowledge, and engagement for all students who transition from the 2-year community college to the 4-year university? Is it more useful for administrators to think of programmatic activities as one general experience or a set of individual experiences, and how does that affect program development?
Multiple	10	SPARC^3^: The future of Associate of Science (Ariyo et al., 2018)	The need for the study precipitated from a program outcome that connected transformational leadership to a STEM program

**Table 5:** Theory families examples

Category	*n*	Example titles	Theory
Critical	1	The intersectional matrix: Rethinking institutional change for URM women in STEM. (Armstrong & Jovanovic, 2017)	Intersectionality theory (Cho et al., 2013)
Organizational literature informed	3	Family Friendly Policies in STEM Departments: Awareness and Determinants (Su & Bozeman, 2016)	Strategy; faculty composition; departmental resources; peer pressure; career aspirations; gender and representative bureaucracy
Multiple organizational theories	1	Leveraging Multiple Theories of Change to Promote Reform: An Examination of the AAU STEM Initiative (Kezar & Holcombe, 2019)	Systems theory; organizational learning; network theory; IT
Neo-institutional Theories	4	Institutional Logics and Institutional Pluralism: The Contestation of Care and Science Logics in Medical Education, 1967–2005 (Dunn & Jones, 2010)	Institutional logics (Thornton & Ocasio, 1999)
No theory	6	n/a	n/a
Organizational learning theories	3	STEM education centers: catalyzing the improvement of undergraduate STEM education (Carlisle & Weaver, 2018)	Organizational learning (Levitt & March, 1988)
Other, non- organizational theories	6	Utilizing factor analysis to inform the development of institutionally contrived experiences to increase STEM engagement (Morgan & Gerber, 2016)	Engagement theory (Astin, 1993)
Theories of organizational change	3	A Systems Model of Innovation Processes in University STEM Education (Porter et al., 2006)	Innovation (Von Hippel, 1998)

### Nuancing organizational learning theory in STEM

To present the results of the second phase of analysis, we highlight an overarching theme and textual evidence for each dimension of organizational change identified from the theoretical framework.

#### Ontological stance

The first dimension focused on whether the study articulated an intentional ontological stance on change. The overarching theme in the articles was that change is vital and desirable in terms of realizing STEM educational interventions. In other words, change is invoked and called for because the status quo does not realize intended goals or outcomes. Studies fell into two categories in terms of how they operationalized this sentiment. One group displayed some gradation in how change is articulated within a process, from explicitly stated (Kezar et al., 2015) to implied based on contextual information from other parts of the study (Su & Bozeman, 2016). For instance, Su and Bozeman (2016) focused on enhancing the representation of female and minority faculty members through structural changes. They acknowledged that “self-assessment is at least an important early step toward more comprehensive changes in departments” (p. 1004). This implicit approach contrasts with explicit and necessary components of the change process outlined in other studies.

Moreover, other studies simply highlighted changing elements without narrating a change process (Ong et al., 2011). In their synthesis of research on factors that contribute to the persistence and success of women of color in STEM fields, Ong et al. (2011) called for transformative and “cultural changes that would improve overall faculty support for and increase the enrollment and retention of minority women” (p. 195). They identified several factors influencing their call for diversifying STEM, including globalization and representation (i.e., external forces), but not a process for this change. However, they recognized the salience “to individual colleges and universities… that support the need to address STEM pedagogy and curriculum for diverse populations as well as research on the relationship between pedagogical changes and cognitive outcomes for women of color” (Ong et al., 2011, p. 198). Ong et al. note the significance of improving the outcomes for women of color in STEM without outlining how to arrive at that desired outcome.

#### Tensions and paradoxes

The second dimension identifies tensions or paradoxical experiences in change processes. Given the nature of paradoxes, it may not be surprising that the dominant theme for this dimension was inconsistency. The tensions or paradoxes identified varied between studies. Most studies did identify tensions/paradoxes in the change process. However, the change was not always nuanced as to what it meant for different stakeholders (Carlisle & Weaver, 2018). Some articles named the role of higher education environments and tensions related to the environment (Rodriguez et al., 2017). Others specified tensions in the research itself versus tensions in the change process (Armstrong & Jovanovic, 2017). The change appeared to bring about a protective nature in wanting to retain previous practices or possession of resources and support, which were concerns specifically emphasized by Kezar (2011). Colbeck (2002) acknowledged that “this planning process is likely to involve considerable conflict as different groups maneuver to ensure their interests are represented” (p. 398). Finally, internal struggles and conflicts often related to infrastructure (Kezar et al., 2015; Lounsbury, 2001). In particular, bottom-up change often had considerable problems with alignment, incentives, involvement, and resources (Kezar et al., 2015).

#### Sustainable change

The third dimension addresses the stability of the change process over time. In examining this dimension, studies described the change as either time-bound or cyclical. Time-bound studies identified a specific period wherein change activities took place, and then the change process came to a resolution. For example, Carlisle and Weaver (2018) described how STEM education “centers [SECs] engage institutions and departments in processes that foster change in undergraduate STEM education, which, if sustained, could lead to the adaptation of traditional norms” (p. 3). This approach implied the changes are not cyclical but end once the centers are a part of institutional norms. Additionally, the purpose of SECs was described as enhancing teaching and learning to broaden participation. In seeking to establish SECs, institutions presumed that establishing centers would provide “a home, as well as resources, for previously funded successful STEM programs and initiatives, thereby contributing to their continuation” (Carlisle & Weaver, 2018, p. 14). This statement again alludes to protecting resources and interests.

The studies that described change as cyclical highlighted adaptive structures to different levels and created the impetus for on-going change. Gehrke and Kezar (2019) suggested that this process should “nurture a regular rhythm for the community, which ensures a continuous cycle of events and involvement opportunities so members can anticipate what is to come through their regular involvement” (p. 849). Kezar and Holcolmbe (2019) pointed out that sustainable change is influenced by multiple levels of change occurring.

#### Stakeholder engagement

The fourth dimension reflects that the change process involves deliberation among stakeholders. The primary theme was that the deliberation process is as much about who is involved as it is about where it happens and how it unfolds. In researching STEM faculty communities of practice, Gehrke and Kezar (2017) were explicitly interested in “focusing on organizationally related outcomes such as department and institutional change” and identifying “the ways in which individual faculty involvement in these communities is related to localized efforts at STEM reform and can thus be leveraged to scale-up reform efforts” (p. 806). Institutional type and structural distinctions also require nuance in the change process deliberation. For example, structural differences might limit collective work (Kezar & Holcombe, 2019) due to timing in collaboration and institutional type contributing to competition for resources.

Reinholz and Apkarian (2018) described “collective goals that attend to, and include, individual goals and concerns”, and a “shared vision for the department can help shape the direction of future change initiatives to align with the needs of individual members as well as build coherence among those goals and ideals” (p. 4). Though some might feel limited in doing collective work, others are deliberately working toward a shared vision. Armstrong and Jovanovic (2017) cited structures or venues that bring URM women together and empower them to promote “community structures” versus deliberation.

#### Network expansion

The fifth dimension explores whether the change process involves expanding the stakeholder’s social network. This theme indicates that the change process is tied to helping stakeholders tap into resources beyond their immediate locus of control. Our findings show this can be prompted in response to external isomorphic pressures or encouraged by what the expanded network could do to support change. The expansion is often related to connections with funding agencies, people with specific expertise, and professional development opportunities. Carlisle and Weaver (2018) ascertained that “a network of partners contributed to STEM education centers’ unique ability to expand institutional capacity” (p. 12). This building of partnerships “consisted of (1) connecting faculty with similar and complementary interests, (2) connecting faculty to available resources, as well as (3) connecting upper administrators to faculty efforts” (p. 12). External agencies identified were related to funding agencies, consultants for their expertise, and professional development opportunities (Gehrke & Kezar, 2017; Henderson et al., 2011). Focusing on external pressure to expand networks comes from isomorphic influences and could relate to intentional efforts versus responding to contextual realities (Su & Bozeman, 2016). Kezar and Holcombe (2019) highlighted what building networks within the institution could do to support the change effort, particularly in terms of espoused versus enacted efforts.

#### Timely access to support and incentives

The sixth and final dimension considers whether the articles narrated timely access to support or incentives. The theme of this dimension focused on structural incentives versus other incentives. Reinholz and Apkarian (2018) indicated that “the inclusion of incentives and rewards for participation in the DAT [Department Action Team] and coordination system as part of the process is aligned with the structural frame” (p. 7). Their structural frame recognizes a culture that includes structures, symbols, power, and people and acknowledges the pressure to maintain power and resources. Rewards and incentives are related to the institutional type and setting and connect to how well-supported faculty and staff feel (Gehrke & Kezar, 2019). The role of stakeholders can help continue to support student success holistically by providing interpersonal support between stakeholders (Rodriguez et al., 2017). It is unclear how timely the support/incentives were within the studies and assumes that support/incentives are ever-present. For example, Carlisle and Weaver (2018) write, “through their scholarship, [STEM education centers] contribute to the knowledge base and provide funding, which adds resources and incentives for the implementation of [evidence-based instructional practices] and educational research” (p. 1).

## Discussion

Much of the research within STEM education focuses on student-level insights (Harper, 2010; Sax et al., 2017) rather than organizational or macro-level dynamics (Bastedo, 2012). In response to our research question, we contend that STEM entities that did not leverage organizational theory and consistently fell into either the “decision trap” identified by Langley et al. (2013) created by ignoring temporal structures or deemed the innovation as threatening as Kezar (2011) suggested. Although not all the innovations focused on URM STEM success, they did attend to innovations that LSAMP is engaging. Hence, the results of our SRL presents two relevant takeaways that complement and challenge the existing knowledge related to the process of scaling an intervention from one context to another and making change sustainable via the institutionalization of structures and supports.

### Can mimetic isomorphism go beyond creating pressure?

A tension we identified in the theoretical framework was that we might be working with “faulty assumptions of change/innovation” (Kezar, 2011, p. 236). Kezar argued that some “practices or ideas that may be perceived as threatening to a community” and, therefore, mutual adaptation is more common (p. 242). Mutual adaption is described as a process whereby an external group or force that views an innovation as beneficial to students persuades and encourages internal groups who may see this innovation as a threat. The findings demonstrate that broadening the impact of innovations that help URM students achieve success in STEM can be viewed as a threat to resources and power within higher education. Creating inclusive practices for STEM URM students has undoubtedly been an external force, often without enough internal champions within various institutions (Ong et al., 2011).

Furthermore, evoking the literature on mimetic isomorphism, the call for a more diverse STEM pipeline has helped to make URM STEM success a priority. Yet ironically, URM STEM success has been so elusive (Ong et al., 2011) that it may allow universities to avoid making real investments and progress in this area. In other words, universities want to imitate their peers in innovating toward URM STEM success. However, if no one achieves this success, there is no real pressure to achieve genuine progress. The work of broadening impacts, specifically concerning URM STEM success, becomes about appearing to scale-up innovations rather than effectively institutionalizing innovative best practices. An interest in the appearance of, rather than a real commitment to innovation, is primarily due to considering innovations a threat to power and resources that could also be influenced by timely decision-making.

### Can organizational frameworks deliver meaningful change in STEM?

Half of the studies that used organizational frameworks focused on organizational learning/theories of change. The other half focused on neo-institutional theories that are more broadly concerned with environmental dynamics. As our second phase of analysis highlighted, some representative studies (Carlisle & Weaver, 2018; Gehrke & Kezar, 2017) attended to both internal and external dynamics of scaling-up an intervention and institutionalized it. This emergent issue highlights the extent to which organizational frameworks help to address whether change efforts are sustainable over time. It is difficult to say definitively since studies in our sample were not designed to answer the question of longevity. The commonality is that change was typically going well at the moment of data collection and journal submission. However, it is much more challenging to determine whether that same success was realized 1 year, 5 years, or 10 years beyond the data collection period and publication time frame.

This raises two additional questions on the utility of organizational frameworks. First, is change meaningful or consequential if it exists for a moment but dissipates over time? Another way of asking the question is how lasting must a change be for it to be meaningful and to what extent does an organizational framework account for the temporal nature of change? The second consideration is how embedded within the organizational framework are insights that prompt reflection or re-engagement with topics after time has passed? As noted, we did not readily identify organizational frameworks or studies that dealt with this issue well beyond Kezar’s (2011) assertion that sustainability of interventions matter.

Consequently, our framework draws attention to the need for stakeholders to reconsider these dimensions in the design and implementation of their change efforts. Hence, we translate these insights into suggestions for STEM organizations within networks that are charged with scaling their innovations and making them sustainable.

### Implications for practice and future research

We distinguish between adaptation and learning in our framework because adaptation refers to defensively changing rather than learning how to apply knowledge and awareness “beyond the immediate event” (Fiol & Lyles, 1985, p. 805). Adapting is not necessarily excluded from organizational learning but is lower-level learning. Fiol and Lyles (1985) contended that higher-level learning “aims at adjusting overall rules and norms rather than specific activities or behaviors” (p. 808). More telling is that lower-level organizational learning is contained within specific areas of an organization, adjusts rules but continues to work within them, and prefers the routine of known and controlled environments.

Unfortunately, STEM initiatives for URMs often function precisely this way. For example, Gomez et al. (2021) researched STEM program directors and described two types of leaders: grassroots leaders and institutional agents. The challenges they identified for each were related to lower-level organizational learning. Grassroots leaders lacked the power and authority to change structures, policies, and resources. Nevertheless, institutional agents also operated within known and controlled environments and often sought to change student behavior rather than their institutions’ rules and norms. Ultimately, they recommended that institutional agents who have the power and authority to change structures become transformation leaders. Doing so requires understanding how their behavior contributes to lower-level rather than higher-level organizational learning. Higher-level organizational learning requires changing mission and direction rather than simply behaviors and rules. Further research could apply lower and higher-level organizational learning to our framework.

The realization that lower-level learning may be acutely linked to URM STEM programs is not unexpected, given what Ray (2019) called the racialization of organizations. Ray asserted that segregation and hierarchy within organizations shape agency, which means there is a disproportionate allocation of resources, specifically for people with minoritized identities. Conversely, Whiteness becomes a “credential providing access to organizational resources, legitimizing work hierarchies, and expanding White agency” (p. 41). This leads to organizations disassociating from even official commitments to equity and inclusion (Ray, 2019). Decoupling from commitments to changing structures, norms, and missions is crucial to organizational higher learning. Leaders and stakeholders should examine how our theoretical framework can be applied in specific ways to consider how racialized organizations influence timely decisions or threats to power. For example, LSAMP alliances could ask if they explicitly understand the process of change and how race influences that process?

## Conclusion

Given the evolving landscape of higher education due to the COVID-19 pandemic, intentional steps that safeguard the STEM ecosystem for URMs (Lord et al., 2019) and access to external resources to support research are critical (Bozeman & Youtie, 2017). We contend that now more than ever, an organizing theory, such as the dimensions of our theoretical framework, is necessary to bring added coherency and credibility to efforts that attend to the needs of marginalized URM students, faculty, and administration who will be differentially impacted by the crisis (Gonzales & Griffin, 2020). Further research and practice are needed to critically, intentionally, and continually examine how this global pandemic can limit how individuals and collective entities engage in organizational learning and change within higher education institutions. By leveraging our key considerations informed by research on this topic to date, we envision a new line of inquiry and practice can be generated to guide how colleges and universities function under this difficult, complex, and challenging time in our history.

## Data Availability

Not applicable.
